# Sodium 4-Phenylbutyrate Reduces Ocular Hypertension by Degrading Extracellular Matrix Deposition via Activation of MMP9

**DOI:** 10.3390/ijms221810095

**Published:** 2021-09-18

**Authors:** Prabhavathi Maddineni, Ramesh B. Kasetti, Bindu Kodati, Sam Yacoub, Gulab S. Zode

**Affiliations:** Department of Pharmacology and Neuroscience, North Texas Eye Research Institute, University of North Texas Health Science Center at Fort Worth, Fort Worth, TX 76107, USA; prabhavathi.maddineni@unthsc.edu (P.M.); ramesh.kasetti@unthsc.edu (R.B.K.); Bindu.Kodati@unthsc.edu (B.K.); sam.yacoub@unthsc.edu (S.Y.)

**Keywords:** glaucoma, trabecular meshwork, ocular hypertension, ER stress, ECM metabolism, MMP, sodium 4-phenylbutyrate

## Abstract

Ocular hypertension (OHT) is a serious adverse effect of the widely prescribed glucocorticoid (GC) therapy and, if left undiagnosed, it can lead to glaucoma and complete blindness. Previously, we have shown that the small chemical chaperone, sodium-4-phenylbutyrate (PBA), rescues GC-induced OHT by reducing ocular endoplasmic reticulum (ER) stress. However, the exact mechanism of how PBA rescues GC-induced OHT is not completely understood. The trabecular meshwork (TM) is a filter-like specialized contractile tissue consisting of TM cells embedded within extracellular matrix (ECM) that controls intraocular pressure (IOP) by constantly regulating aqueous humor (AH) outflow. Induction of abnormal ECM deposition in TM is a hallmark of GC-induced OHT. Here, we investigated whether PBA reduces GC-induced OHT by degrading abnormal ECM deposition in TM using mouse model of GC-induced OHT, ex vivo cultured human TM tissues and primary human TM cells. We show that topical ocular eye drops of PBA (1%) significantly lowers elevated IOP in mouse model of GC-induced OHT. Importantly, PBA prevents synthesis and deposition of GC-induced ECM in TM. We report for the first time that PBA can degrade existing abnormal ECM in normal human TM cells/tissues by inducing matrix metalloproteinase (MMP)9 expression and activity. Furthermore, inhibition of MMPs activity by chemical-inhibitor (minocycline) abrogated PBA’s effect on ECM reduction and its associated ER stress. Our study indicates a non-chaperone activity of PBA via activation of MMP9 that degrades abnormal ECM accumulation in TM.

## 1. Introduction

Glucocorticoids (GCs) are one of the widely prescribed medications to treat various autoimmune and inflammatory conditions due to their potent anti-inflammatory and immunosuppressive actions [1]. Despite its numerous benefits, prolonged GC therapy can cause ocular adverse effects including ocular hypertension (OHT) and, if left untreated, it can lead to iatrogenic open-angle glaucoma [2,3,4]. Interestingly, the morphological and clinical manifestation of GC-induced glaucoma are similar to primary open angle glaucoma (POAG), the most common form of glaucoma accounting for ~70% of the total cases [3,5,6,7]. Indeed, both POAG and GC-induced glaucoma are associated with increased intraocular pressure (IOP), which leads to progressive loss of retinal ganglion cell (RGC) axons and irreversible loss of vision [1,8,9,10,11]. Therefore, unraveling and targeting the underlying molecular mechanisms of GC-induced OHT are of great interest to develop novel therapeutics for glaucoma. Under normal conditions, trabecular meshwork (TM), located in the iridocorneal angle maintains IOP homeostasis by constantly regulating aqueous humor (AH) outflow. Cellular dysfunction in TM tissue can lead to reduction of AH outflow and elevation of IOP. Like POAG, ocular or systemic administration of GCs induces morphological and biochemical changes in TM, leading to TM dysfunction and OHT [12,13,14,15,16,17]. Depending on the duration of GC therapy, its potency, and the route of administration, the susceptibility of GC- induced OHT varies from 5% to 30% in normal population and 92% in POAG patients [3,7,8,9].

An altered extracellular matrix (ECM) metabolism and chronic endoplasmic reticulum (ER) stress are known to be involved in TM dysfunction [15,18]. Evidence from human perfusion culture studies and mouse models of glaucoma demonstrated that chronic GC treatment leads to TM pathology as evident from thickening of trabecular beams, decreased intertrabecular spaces, and increasing synthesis and deposition of ECM molecules and formation of crosslinked actin networks [11,14,19]. In addition, an imbalance of matrix metalloproteinases (MMPs) and their endogenous tissue inhibitors (TIMPs) with a shift toward raised TIMPs levels was observed in glaucomatous AH [20,21,22,23,24]. It is becoming increasingly evident that the extracellular milieu is important in maintaining TM integrity [16]. Moreover, TM cells could sense and translate the intrinsic biophysical properties of ECM into intracellular signals and control their own gene transcription, protein expression, and cell behavior [25]. Previously we have shown that GC-induced altered ECM metabolism triggers chronic ER stress in TM cells [12,13], highlighting the existence of cross talk between abnormal ECM and chronic ER stress in glaucomatous TM pathology. It has been also reported that TM cells are more susceptible to ER stress and its associated apoptotic cell death compared to the other ocular cells [12]. All these pathological changes may lead to TM tissue stiffness with reduced contractility and cellular dysfunction, and further contribute to AH outflow facility obstruction and OHT. 

Although increased ECM deposition is a major feature of the glaucomatous TM pathology, none of current treatments directly target this pathology to lower elevated IOP in glaucoma. Previous studies from our laboratory have shown that topical ocular eye drops of sodium 4-phenylbutyrate (PBA) rescued mouse models of GC or MYOC-induced glaucoma (*Tg-MYOC^Y437H^* mice) [18,26]. PBA is an aromatic short-chain fatty acid, approved by the U.S. Food and Drug Administration (FDA) for clinical use in patients with urea cycle disorders and it has a good safety profile. Although PBA reduces elevated IOP by restoring TM function, the mechanism of action of PBA is not yet completely understood. In *Tg-MYOC^Y437H^* mice, which express misfolded mutant myocilin, PBA acts as a chaperone and enhances the proper folding and secretion of mutant myocilin and thereby reduces ER stress in the TM [26]. PBA has also been shown to reduce protein mis-localization and ER stress in other diseases via its chaperonin activity [27,28]. Other studies have also shown that PBA exerts non-chaperonin activities likely due to its histone deacetylase (HDAC) inhibition [28]. However, it is not understood how PBA reduces GC-induced ER stress and IOP elevation. 

In the present study, we explored whether PBA rescues GC-induced OHT via degradation of abnormal ECM. Using a mouse model of GC-induced OHT, primary human TM cells, and ex vivo human corneoscleral segments culture model, we show that PBA prevents de novo synthesis of ECM and reduces existing ECM deposition, lowering elevated IOP in mouse model of GC-induced OHT. Importantly, we report for the first time that PBA degrades existing ECM deposition via activation of MMP9.

## 2. Results

### 2.1. PBA Reduces Dexamethasone 21-Acetate (Dex)-Induced OHT and Decreases ECM Deposition and ER Stress in TM Tissue

**Figure 1:**
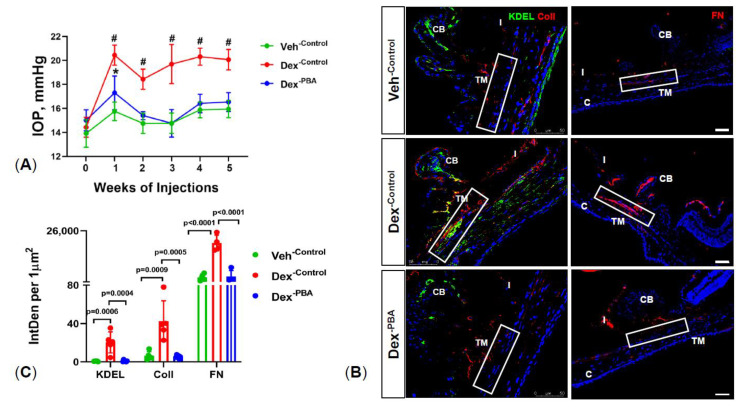
PBA prevents Dex-induced IOP elevation and abnormal ECM deposition in mouse TM: Three-month old C57BL/6J mice were injected bilaterally once a week for 5 weeks with either Veh or Dex and applied 6 µL of topical ocular eye drops of either water (control) or PBA (1%) twice a day throughout the study period. (**A**) Dex-injected mice receiving control eye drops (Dex^-Control^) showed significant IOP elevation compared to Veh-injected mice receiving control eye drops (Veh^-Control^). Topical ocular eye drops of PBA (1%) significantly lowered IOP in Dex-injected mice (Dex^-PBA^) compared to Dex^-Control^ mice. Data are shown as mean  ±  SD (*n*  =  8 to 10 eyes; 2–WAY ANOVA with multiple comparison; * indicates *p*  = 0.02; # indicates *p*  <  0.0001). (**B**) Immunohistochemical images showing expression of KDEL and ColI (left panel) and FN (right panel) in mouse TM tissues (highlighted by a rectangle white box) (Scale bar is 50µm). (**C**). Densitometric analysis showing increased expression of ER stress (KDEL) and ECM proteins (ColI and FN) in TM tissues of Dex^-Control^ mice. Additionally, 1% PBA topical ocular eyedrops significantly reduced ER stress and ECM markers in the TM tissues of Dex^-PBA^ mice group. Data are shown as mean  ±  SD (*n* = 4, ONE WAY ANOVA with multiple comparison). CB: ciliary body; I: iris; C: cornea.

We have previously shown that weekly periocular injections of Dex leads to OHT [14] and glaucoma in mice [11]. We sought to determine whether PBA reduces elevated IOP in our recently developed mouse model of Dex-induced glaucoma [11]. First, 3-month-old C57BL/6J mice were injected with either Veh or Dex via periocular route and treated with 6 μL topical ocular eyedrops of water or PBA (1%). IOPs were monitored weekly before and after treatments. As shown in Figure 1A, Dex-injected mice with topical ocular eye drops of water (denoted as Dex^-Control^) showed sustained and significant IOP elevation compared to Veh^-Control^ mice. Starting from the first week of treatment, Dex^-Control^ mice demonstrated a mean IOP difference of ~3.5 to 4.5 mmHg over Veh^-Control^ mice group. Interestingly, topical ocular PBA eye drops (given twice a day) significantly reduced Dex-induced IOP (Dex^-PBA^ mice). We observed no differences between Dex^-PBA^ mice and Veh^-Control^ mice groups. We next explored whether PBA reduces abnormal ECM accumulation and ER stress in TM tissues. Immunostaining for collagen I (ColI), fibronectin (FN), and ER stress marker (KDEL, recognizes GRP78 and GRP94) was performed on mouse anterior segment tissues from different treatment groups (Figure 1B). Consistent with our previous studies [11,14], an increased expression of ColI, FN, and KDEL was observed in the TM tissues of Dex^-Control^ mice compared to Veh^-Control^ mice (Figure 1B,C). Importantly, Dex^-PBA^ mice showed dramatically decreased ColI, FN, and KDEL in TM (Figure 1B,C). These data clearly indicate that PBA effectively lowers IOP and reduces abnormal ECM deposition and ER stress in Dex-treated mice.

### 2.2. PBA Reduces Dex-Induced Synthesis of ECM Proteins in Primary Human TM Cells

**Figure 2:**
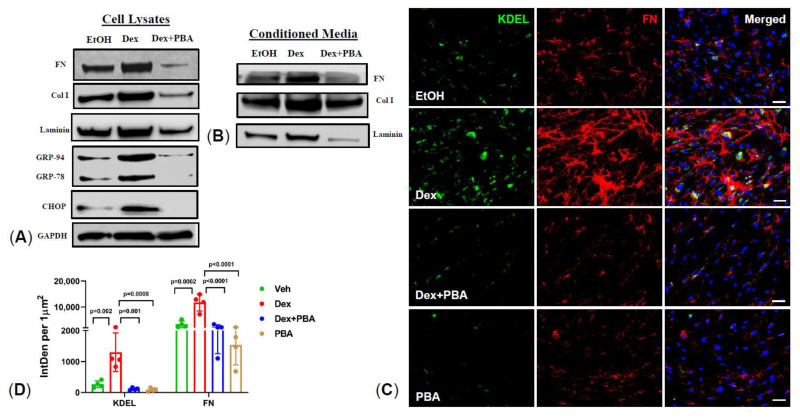
PBA prevents Dex-induced ECM synthesis and ER stress in primary human TM cells: Primary human TM cells were treated with ethanol, Dex (100 nM), and Dex + PBA (5 mM) for 7 d and ECM and ER stress markers were determined in cellular lysates (**A**) and conditioned media (**B**) by Western blotting and immunostaining (**C**,**D**). Increased levels of ECM (FN, ColI, and laminin) and ER stress (GRP-94, GRP-78, and CHOP) markers were observed in Dex-treated cells, and PBA treatment reduced Dex-induced ECM and ER stress in primary TM cells (**A**,**B**). Representative immunostaining images of KDEL and FN (**C**), and their intensity measurements (**D**) further confirmed that PBA significantly reduces Dex-induced ER stress and ECM synthesis in primary human TM cells. Data are shown as mean  ±  SD (*n* = 3–4, ONE WAY ANOVA with multiple comparison, scale bar 50 µm).

As we reported earlier [12], Dex induction of de novo synthesis of ECM proteins in TM is associated with IOP elevation. We therefore examined whether PBA reduces de novo ECM synthesis and restores ER homeostasis in human TM cells. Primary human TM cells (*n*  =  3 cell strains) were treated with either ethanol or Dex or Dex plus PBA for 7 d. Total cell lysates and conditioned media were collected and subjected to Western blot analysis of major ECM and ER stress markers. As shown in Figure 2A**,** PBA effectively decreased Dex induced ECM synthesis including FN, ColI, and laminin (major ECM components of TM) and also reduced KDEL and CHOP (chronic ER stress) induction in TM cells. Additionally, PBA reduced Dex-induced secreted levels of FN, ColI, and laminin in the conditioned media (Figure 2B). Moreover, an increased expression and co-localization of FN with KDEL in Dex-treated cells was dramatically reduced upon PBA co-treatment (Figure 2C,D). These data demonstrate that PBA prevents Dex induced ECM synthesis and induction of ER stress in primary human TM cells.

### 2.3. PBA Reduces Dex Induced ECM Deposition in Primary Human TM Cells

**Figure 3:**
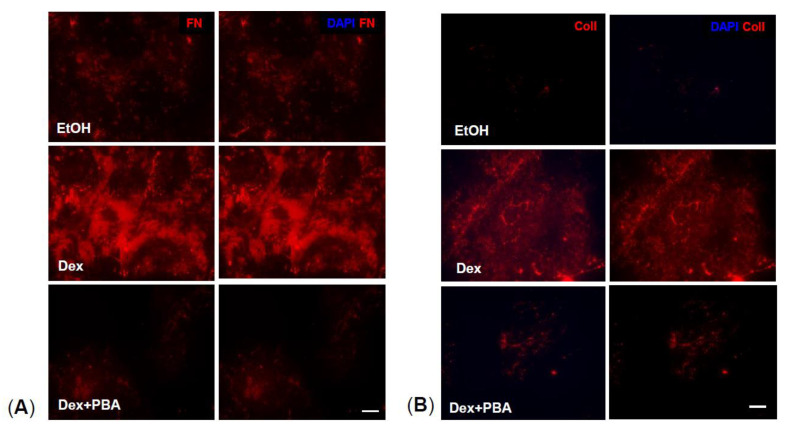
PBA reduces Dex-induced abnormal ECM deposition: Primary human TM cells were grown to 80% confluency on a 4-well glass chamber slides and treated with either ethanol, Dex (100 nM) and Dex + PBA (5 mM) for 7 d. Following decellularization with Tritox-100 and NH_4_OH, wells were stained with ECM markers (FN and ColI) and DAPI. Absence of DAPI further confirmed the successful decellularization process. An increased extracellular deposition of FN (**A**) and ColI (**B**) was observed in Dex-treated cells compared to Veh-treated cells. However, in Dex + PBA treated cells, a prominent decline in ECM deposition was observed (Scale bar 50 µm).

We next examined whether PBA also reduces ECM deposition. Primary human TM cells were treated with either ethanol or Dex or Dex plus PBA for 7 d. ECM from different treatment conditions were obtained by decellularization process using Triton X-100 and NH_4_OH, and further used for immunostaining analysis. We observed an increased expression of FN (Figure 3A) and ColI (Figure 3B) in Dex-derived ECM compared to ethanol-derived ECM. Interestingly, Dex plus PBA derived ECM demonstrated significantly reduced FN (Figure 3A) and ColI (Figure 3B) levels, indicating that PBA reduces Dex-induced ECM deposition in primary human TM cells.

### 2.4. PBA Prevents Abnormal ECM Induced ER Stress in Primary Human TM Cells

**Figure 4:**
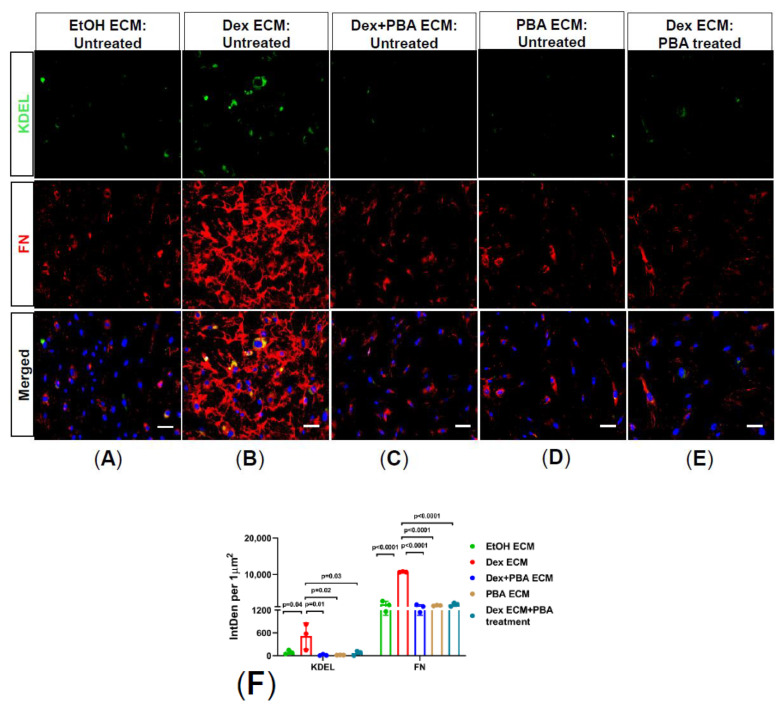
PBA degrades ECM deposition and prevents ER stress in primary human TM cells: Primary human TM cells were grown to 80% confluency on the chamber slides, and then treated with either ethanol (control) or Dex (100 nM) or Dex plus PBA (5 mM) or PBA for 7 d and subjected to decellularization. Following complete decellularization, fresh primary human TM cells were re-grown on decellularized ECM for another 5 d, left either untreated (**A**–**D**) or treated with PBA (5 mM) (**E**). Representative immunostaining images (**A**–**E**) and their intensity measurements (**F**) confirmed that abnormal ECM derived from Dex-treated TM cells (**B**) induces ER stress (KDEL) compared to TM cells grown in control-derived ECM (**A**). Primary TM cells re-grown on Dex + PBA (**C**) and PBA (**D**) derived ECM did not trigger ER stress compared to Dex-induced ECM deposition (**B**). Interestingly, freshly regrown TM cells on Dex-derived ECM showed no signs of ER stress (KDEL) and degradation of ECM (FN) upon PBA treatment (**E**). (*n* = 3 strains, scale bar 50µm, ONE WAY ANOVA with multiple comparison).

ECM has profound effects on TM and changes in ECM rigidity influence TM cell behavior leading to cellular dysfunction and OHT. We therefore sought to explore whether PBA mediated reduction of abnormal ECM also prevents ER stress using primary human TM cells plated on decellularized Dex-derived ECM. Firstly, primary human TM cells were treated with either ethanol or Dex or Dex plus PBA or PBA alone for 7 d and obtained decellularized ECM from all the treatment conditions. Furthermore, fresh primary human TM cells were plated on ethanol or Dex or Dex plus PBA or PBA-derived decellularized ECM and cultured for an additional 5 d with or without PBA treatment. As shown in Figure 4, TM cells which were plated on the abnormal Dex-derived ECM (Figure 4B) induced ER stress (KDEL) compared to cells plated on control ECM (Figure 4A). Consistently, immunostaining revealed decreased FN and KDEL expression in TM cells plated on Dex plus PBA (Figure 4C) and PBA-derived ECM (Figure 4D), indicating that PBA reduces Dex-induced abnormal ECM deposition and also prevents ER stress in TM cells. Moreover, when TM cells were re-plated on abnormal Dex-derived ECM and then treated with PBA (Figure 4E), PBA decreased FN and KDEL staining indicating that PBA degrades the pre-existing abnormal ECM and rescues primary TM cells from abnormal ECM induced ER stress.

### 2.5. PBA Rescues Primary TM Cells from Exogenous Cellular Fibronectin (cFN) Induced ER Stress

**Figure 5:**
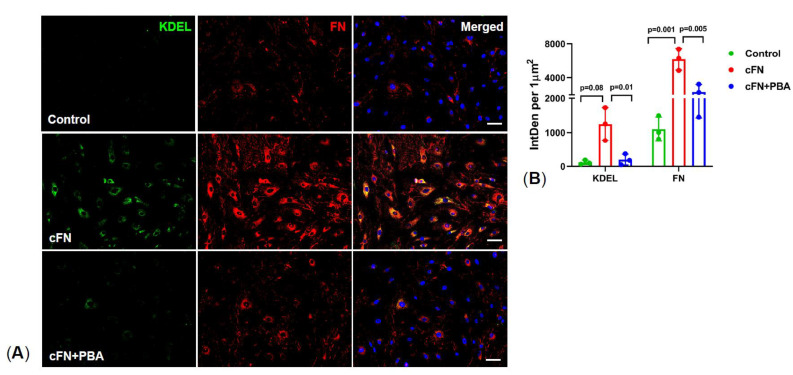
PBA degrades cellular fibronectin and prevents ER stress in primary human TM cells: Primary human TM cells were grown to 80% confluency on 4-well glass chamber slides, treated with either cFN (10  µg/mL) or cFN + PBA for 5 d, and immunostained for FN and KDEL (ER stress). Representative images (**A**) and analysis (**B**) confirmed that PBA degraded cFN and also prevented ER stress associated with cFN in primary human TM cells. (*n* = 3 strains, scale bar 50 µm, ONE WAY ANOVA with multiple comparison).

Using exogenous cFN, we further investigated whether PBA degrades ECM and prevents ER stress in primary human TM cells. cFN is an insoluble isoform of FN, which forms fibril networks and regulates ECM-cell interactions. Primary human TM cells were treated with either cFN or cFN plus PBA for 5 d and analyzed for FN and KDEL by immunostaining. As shown in Figure 5, treatment of primary human TM cells with cFN induced ER stress as evident from increased KDEL, suggesting that an altered ECM deposition can trigger ER stress in TM cells. Interestingly, cFN plus PBA treated cells showed reduced expression of both FN and KDEL, indicating PBA degrades the exogenous cFN and rescues primary TM cells from ER stress.

### 2.6. PBA Degrades ECM by Upregulating MMP9 Gene and Protein Expression

**Figure 6:**
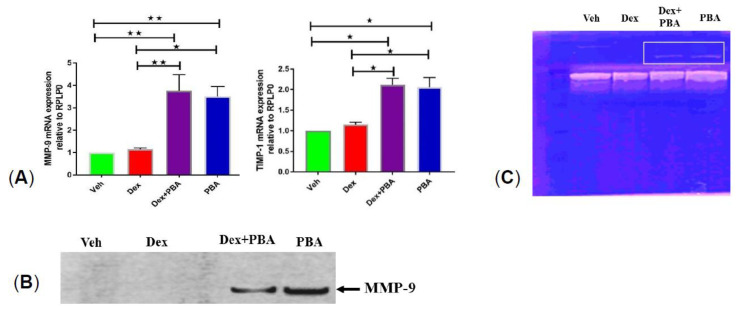
PBA induced MMP9 expression and activity: **A**) relative mRNA expression level of *MMP9* and *TIMP-1* in transformed human GTM-3 cells, treated with either ethanol (Veh) or Dex (100 nM) or Dex + PBA (5 mM) or PBA for 24 h. Conditioned media was subjected to Western blotting (**B**) and gelatin zymography (**C**). We observed a prominent increase in protein expression (**B**) and the enzymatic activity (**C**) of MMP9 (represented by white box) in the conditioned media derived from Dex + PBA and PBA treated TM cells, but not in ethanol and Dex treated GTM-3 cells. (*n* = 3, ONE WAY ANOVA with multiple comparison, ★ indicates *p* < 0.05, ★★ indicates *p* < 0.001).

Given that PBA degrades the abnormal or glaucomatous ECM, we next examined whether PBA has any effect on MMPs. Here, we focused on MMP9 axis because it is one of the main MMPs which was shown to be reduced in human glaucomatous AH [29]. Moreover, genetic association studies have revealed a significant association of MMP9 (−1562C > T) polymorphism with human POAG patients [30]. Transformed GTM-3 cells were treated with ethanol or Dex or Dex plus PBA or PBA for 24 h and mRNA expression of *MMP9* and its endogenous inhibitor *TIMP1* was analyzed via qPCR. We observed an increased mRNA expression of both *MMP9* and its tissue inhibitor *TIMP1* in Dex plus PBA and PBA-treated cells compared to ethanol or Dex-treated cells (Figure 6A). Although both *MMP9* (~4 fold) and its tissue inhibitor *TIMP1* (~2 fold) were upregulated, we observed a shift toward raised *MMP9* levels with PBA (Figure 6A) treatment. We further examined the expression and enzymatic activity of MMP9 in conditioned media by Western blot and gelatin zymography respectively. We observed an increased level of active MMP9 by Western blot (Figure 6B) and induction of MMP9 enzymatic activity (Figure 6C) in Dex plus PBA and PBA treated TM cells. These data suggest that PBA may degrade existing ECM by upregulating MMP9 protein and enzymatic activity.

### 2.7. Inhibition of MMPs Abrogates PBA’s Effect on ECM Degradation and Its Associated ER Stress

**Figure 7:**
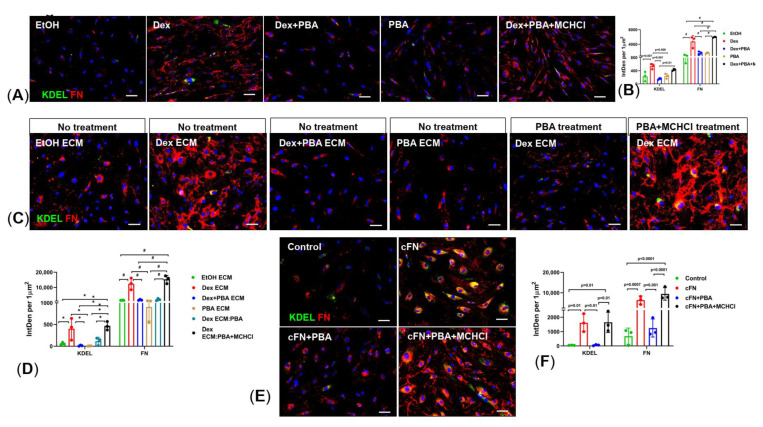
Inhibition of MMPs alleviates PBA’s effect on abnormal ECM degradation and its associated ER stress: (**A**,**B**) Primary human TM cells were treated with either ethanol, Dex (100 nM), Dex + PBA (5 mM), or PBA and Dex + PBA + MCHCl (200 µM) for 7 d. PBA significantly reduced Dex-induced ECM deposition and its associated ER (Dex + PBA) in TM cells. However, in the presence of MMP inhibitor MCHCl, the effect of PBA was abrogated to reduce ECM deposition and ER stress in cells treated with Dex + PBA + MCHCl. (**C**,**D**) Primary human TM cells were re-grown on decellularized ECM derived from either ethanol or Dex (100 nM) or Dex + PBA (5 mM) or PBA. The regrown TM cells on Dex-derived ECM showed reduced ER stress (KDEL) and degradation of ECM (FN) upon PBA treatment (Dex ECM: PBA treatment) compared to untreated regrown TM cells on Dex-derived ECM (Dex ECM). However, the regrown TM cells on Dex-derived ECM showed prominent increase in ER stress and ECM, when the cells were co-treated with PBA and MCHCl (200 µM) (Dex ECM: PBA + MCHCl treatment). (**E**,**F**) PBA reduced ER stress (KDEL) by degrading cFN in TM cells, treated with cFN (10 µg/mL) + PBA (5 mM) compared to cFN alone treated cells. However, in the presence of MCHCl (200 µM), PBA unable to degrade cFN and thus observed induction of ER stress in TM cells, treated with cFN + PBA + MCHCl. (*n* = 3, ONE WAY ANOVA with multiple comparison, * indicates *p* < 0.05, # indicates *p* < 0.0001, scale bar 50 µm).

We next examined whether inhibition of MMPs using minocycline hydrochloride (MCHCl) abrogates protective effects of PBA on ECM deposition and degradation. For this, primary human TM cells were treated with either ethanol or Dex or Dex plus PBA or PBA or Dex plus PBA plus MCHCl for 7 d and examined ECM deposition (Figure 7A,B). We observed a significant reduction in ECM deposition and its associated ER stress in TM cells, treated with Dex plus PBA compared to Dex alone treated TM cells. However, in the presence of MCHCl, the effect of PBA on Dex-induced ECM deposition and ER stress was abrogated (Dex + PBA + MCHCl) (Figure 7A,B). In addition, primary human TM cells were plated on either Dex-derived decellularized ECM or exogenous cFN and further treated with PBA along with MCHCl. As shown in Figure 7C,D, primary human TM cells plated on Dex-derived ECM showed reduced ER stress (KDEL) and degradation of ECM (FN) upon PBA treatment (Dex ECM: PBA treatment) compared to untreated TM cells which were plated on Dex-derived ECM (Dex ECM). However, the regrown TM cells on Dex-derived ECM showed prominent increase in ER stress and ECM, when the cells were co-treated with PBA and MCHCl (Dex ECM: PBA + MCHCl treatment). Similarly, in the absence of MCHCl, PBA had degraded the exogenous cFN and there by significantly reduced the induction of ER stress in TM cells. However, in the presence of MMP inhibitor MCHCl, PBA unable to degrade exogenous cFN and reduce ER stress in TM cells. (Figure 7E,F).

### 2.8. Effect of PBA on Dex Induced ECM Deposition and ER Stress in Ex Vivo Cultured Human Corneoscleral Segment Tissue

**Figure 8:**
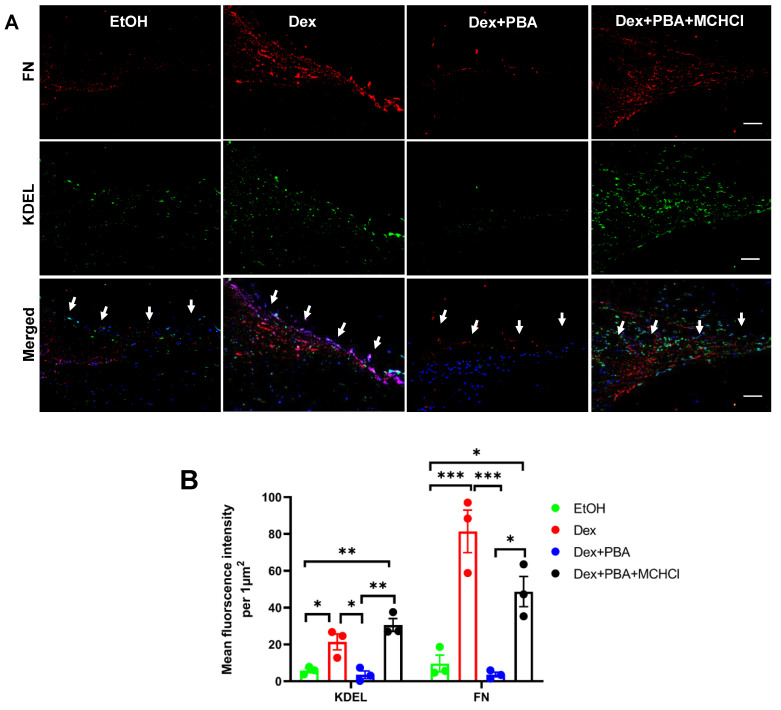
Effect of PBA on Dex induced ECM deposition and ER stress in ex vivo cultured human corneoscleral segment tissue: Human corneoscleral segments with an intact TM rim were dissected into four equal quadrants and each quadrant was cultured and treated with either ethanol or Dex (100 nM) or Dex + PBA (5 mM) or Dex + PBA + MCHCl (200 µM) for 7 d. Immunostaining (**A**) and densitometric analysis of (**B**) FN and KDEL on ex vivo cultured human corneoscleral segments. (*n* = 3, ONE WAY ANOVA with multiple comparison, * indicates *p* < 0.05, ** indicates *p* < 0.001, *** indicates *p* < 0.0001, scale bar 50 µm, the white arrow represents the TM region).

Using the ex vivo cultured human corneoscleral segments, we further investigated whether PBA reduces Dex-induced ECM and ER stress in human donor eyes. Human corneoscleral segments with intact TM rim were dissected into four equal quadrants and treated separately with either ethanol or Dex or Dex plus PBA or Dex plus PBA plus MCHCl for 7 d. Immunohistochemical analysis revealed an increased FN (ECM marker) and KDEL (ER stress marker) levels in Dex-treated corneoscleral quadrants compared to the vehicle-treated controls (Figure 8). Interestingly, PBA treated corneoscleral quadrants reduced Dex-induction of FN and KDEL in human TM tissues (Figure 8). However, co-treatment with MCHCl abrogated this protective effect of PBA and resulted in an increased ECM deposition and ER stress. (Figure 8). 

## 3. Discussion

Considering a widespread use of GCs for the treatment of various ocular conditions, GC-induced OHT is a serious clinical problem. It has been reported that transactivation of GC-receptor is responsible for GC-induced OHT and glaucoma [31]. Alternatively, studies with new selective GC receptor agonists with reduced transactivation have been initiated to avoid the adverse effects of GCs [32]. Understanding and targeting GC-induced OHT is of great interest since it mimics POAG including reduced AH outflow and TM cell dysfunction [2,7,10,11,14,33,34,35]. In this study, we demonstrated PBA’s therapeutic usefulness to prevent GC-induced OHT and identified its non-chaperonin activity by modulating ECM metabolism. Furthermore, we demonstrate that PBA can degrade the pathological ECM in TM by upregulating MMP9 gene and its enzymatic activity.

TM is the major component of the conventional outflow pathway and acts as a molecular sieve consisting of TM cells embedded within ECM. TM maintains normal IOP homeostasis by providing resistance to AH outflow. In POAG or GC-induced glaucoma, this resistance to AH is increased, leading to elevation of IOP. Several lines of evidence have suggested that increased deposition of ECM proteins and induction of chronic ER stress are associated with increased outflow resistance at the TM [12,15]. Moreover, ECM deposited by glaucomatous TM cells are stiffer than those deposited by non-glaucomatous TM cells [25]. It is becoming increasingly evident that the abnormal ECM deposition alters the TM integrity and rigidity. Particularly, studies using atomic force microscopy and OCT Imaging have revealed that abnormal ECM deposition contributes to glaucomatous TM stiffness in both POAG and GC-induced glaucoma [17,36,37,38,39] and also alters intracellular signaling of the TM cell itself [25,34]. It is therefore critical to develop novel therapies aimed at degrading existing ECM deposition in TM. Unfortunately, none of the current IOP lowering treatments target abnormal ECM accumulation in TM. To our knowledge, this is the first study that targets ECM synthesis and deposition in TM to reduce OHT. Importantly, PBA can degrade the exiting aberrant ECM and further inhibit ER stress induction in TM cells. The ability of PBA to degrade existing abnormal ECM deposition is critical since most glaucoma patients are diagnosed at relatively late stage with substantial pathology to TM including ECM accumulation. It is likely that PBA’s ability to reverse existing abnormal ECM accumulation will greatly restore TM’s function to maintain IOP homeostasis.

In the non-glaucomatous TM cells, ECM undergoes continual remodeling to maintain normal AH outflow facility. MMPs, zinc-dependent endopeptidases, and their endogenous inhibitors TIMPs are involved in this ECM remodeling and turnover. MMPs are secreted as proenzymes and activated extracellularly. The activity of the MMPs is regulated mainly by TIMPs (TIMP1, 2, 3, and 4) which binds MMPs in a 1:1 stoichiometry and cause reversible inhibition of MMPs and hence the ratio of MMP:TIMP often determines the extent of ECM turnover [24,40]. An altered expression of MMPs and imbalances in the MMP/TIMP ratio have been associated with abnormal fibrillary ECM accumulation in glaucomatous TM [20,21,23,24,41]. Using human organ culture perfusion system, Bradle et al. reported an increased AH outflow with the addition of exogenous purified MMPs and decreased outflow with MMP inhibitors [42]. Trabeculoplasty, which is used clinically to ameliorate the IOP, also induces dramatic and sustained MMP expression, specifically in the TM, and decreases outflow resistance [43]. Several studies in POAG patients have also shown the association between decreased MMP9 activity in the TM with the development of OHT [44,45,46]. MMP9, a type IV collagenase or gelatinase B, plays a major role in the degradation of major ECM components of TM including FN, collagens, and laminin [47]. Similar to POAG, exposure of TM organ cultures to Dex also resulted in decreased activity of MMP9 [22,48]. Moreover, an aberrant collagen deposition in TM and OHT was reported in MMP9 knockout mice, suggesting that ECM remodeling by MMP9 is required to maintain IOP homeostasis [46]. Consistent with these findings, we have also observed downregulation of MMP9 in human corneoscleral segments as well as in primary human TM cells upon Dex treatment. Interestingly, PBA enhances the enzymatic activity of MMP9 and reverses the GC-induced ECM deposition and TM pathology. Although PBA increases both *MMP9* and *TIMP1* at transcription level, the ratio of *MMP-9* to *TIMP1* is higher and thus regulates aberrant ECM remodeling in the TM.

Given that MMPs regulate ECM turnover within the outflow tissues and contribute to increased outflow facility, various pharmacological and viral vectors with enhancing MMP activity are currently under clinical investigation as a hypotensive medication to treat glaucoma. It has also been reported that sigma1 receptor and its agonists modulate the IOP through the MMP-9 regulation [49,50]. Prostaglandin analog/prostamide (PGA) including latanoprost, bimatoprost, and travoprost, the current glaucoma treatment regimen, has been shown to increase MMP expression in TM and ciliary body, leading to tissue remodeling and enhanced AH outflow [24,40]. The IOP lowering efficacy of individual PGAs depends on their MMP activation profiles [51]. Like PGA, PBA also acts as hypotensive medication and reduces IOP partly by upregulating MMP9 expression and its enzymatic activity. Importantly, we did not notice either stromal ECM remodeling or corneal thinning with topical ocular eyedrops of PBA. Moreover, PBA is pleiotropic and acts as histone deacetylase inhibitor [28], ammonia scavenger [27], and chemical chaperone [18,26,52]). Therefore, it has potentially favorable effects on various pathologic conditions including cancer, genetic metabolic syndromes, and neuropathies. Depending on the cell type or disease phenotype, PBA exerts its specific effect and has good safety profile. Previously, Zode et al. reported that PBA reduces glaucomatous phenotypes by reducing myocilin accumulation in *Tg-MYOC^Y437H^* mice [26] and chronic ER stress in in murine GC-induced glaucoma [18].

In conclusion, we have demonstrated that PBA reduces GC-induced OHT by reducing ECM synthesis and deposition in TM. We further presented evidence that PBA upregulates MMP9 activity, which degrades existing abnormal ECM deposition in TM. Our study highlights both chaperonin and non-chaperone activity of PBA and suggests that PBA can be an attractive treatment for glaucoma via targeting both abnormal ECM accumulation and chronic ER stress in the TM.

## 4. Methods and Materials

### 4.1. Experimental Animals

Three-month-old male C57BL/6J mice were obtained from the Jackson Laboratory (Bar Harbor, ME, USA). Mice were fed standard chow ad libitum and housed in 12 h light/12 h dark conditions with proper temperature (21 °C to 26 °C) and humidity (40% to 70%). All the experimental procedures used in this study were conducted in accordance with and adherence to the ARVO Statement for the Use of Animals in Ophthalmic and Vision Research, and the experimental protocol was approved by the Institutional Animal Care and Use Committee (IACUC) of the University of North Texas Health Science Center (UNTHSC) (Protocol #: IACUC-2018–0032).

### 4.2. Antibodies and Reagents

Antibodies and reagents were purchased from the following sources: fibronectin (catalog # Ab2413, Abcam, Cambridge, MA, USA), KDEL (catalog # NBP1–97469, Novus Biologicals, Littleton, CO, USA), collagen I (catalog # NB600–408, Novus Biologicals, Littleton, CO, USA), laminin (catalog # NB300–144, Novus Biologicals, Littleton, CO, USA), CHOP (catalog # 13172, Novus Biologicals, Littleton, CO, USA), and GAPDH (catalog # 3683, Cell signaling technology, Danvers, MA, USA), dexamethasone-21-acetate (Spectrum Chemicals, New Brunswick, NJ, USA), minocycline hydrochloride (Enzo Life Sciences, Inc., New York, NY, USA), and PBA (Scandinavian formulas, PA, USA).

### 4.3. GC-Induced Mouse Model of OHT

As described previously [11,14], mouse model of GC-induced OHT was generated using weekly periocular injections of a potent GC, Dexamethasone-21-Acetate (Dex). Briefly, 3-month-old C57BL/6J mice were bilaterally injected with 20 μL/eye of either Vehicle (Veh) or Dex (200 µg/eye) under anesthetic conditions (isoflurane (2.5%); oxygen (0.8 L/min)) for 5 weeks. Weekly IOP measurements were performed to ensure OHT.

### 4.4. IOP Measurements

IOP measurements were recorded in a masked manner using a rebound TonoLab tonometer (Colonial Medical Supply, Franconia, NH, USA). Under isoflurane anesthetic (isoflurane (2.5%); oxygen (0.8 L/min)) conditions, daytime IOPs were monitored once a week for 5-weeks in Veh and Dex injected mice. At each time point, an average of five to six IOP readings per eye were taken. IOP measurements were completed within 4 to 5 min to avoid the effect of isoflurane on IOP.

### 4.5. Topical Ocular Eye Drops of PBA

As described previously [18,26], topical ocular eye drops of 1% PBA were applied twice a day. Briefly, C57BL/6J mice were injected bilaterally with either Veh or Dex via periocular route. One group of each Veh and Dex-treated mice received water as a control eye drops (6  μL/eye) while other group received 1% PBA eye drops (6  μL/eye) throughout the study period, starting from the day of injections.

### 4.6. Human Primary TM Cells

Primary human TM cells from normal donor eyes (*n* = 3–4) were isolated and characterized as described previously [53]. Primary TM cell strains (between passages 5 and 7) were seeded in either 6-well plate or chamber slides and grown to ~80% confluence in DMEM-low glucose medium (Sigma, St. Louis, MI, USA), supplemented with 10% fetal bovine serum (Atlas Biologicals, Fort Collins, CO, USA), L-glutamine (Gibco, Life technologies, Grand Island, NY, USA), and Pen-strep (Gibco, Life technologies, Grand Island, NY, USA). Primary TM cells were treated with either 0.1% ethanol (control) or Dex (100 nM) alone (Sigma-Aldrich Corp., St. Louis, MO, USA) or Dex (100 nM) plus PBA (5 mM) or PBA (5 mM) alone, and further grown to 100% confluency for 7 d. In some instances, cells were also treated with MMP inhibitor minocycline hydrochloride (MCHCl, 200 µM). For Western blot analysis, both cell lysates and conditioned medium were collected. For immunostaining, cells were fixed in 4% paraformaldehyde and stained with appropriate antibodies.

### 4.7. Exogenous Cellular Fibronectin (cFN) Treatment

To analyze the effect of PBA on fibronectin degradation, TM cells were treated with 10 µg/ml cellular fibronectin (F2518; Sigma-Aldrich Corp., St. Louis, MO, USA) for 48 h and incubated with or without 5 mM PBA for an additional 5 d. Cells were fixed and analyzed for fibronectin and ER stress by immunostaining.

### 4.8. Ex Vivo Human Corneoscleral Segments Cultures

Human donor eyes were obtained from the Willed Body Program (UNTHSC, Fort Worth, TX, USA) in accordance with Declaration of Helsinki guidelines. The study design and conduct complied with all the relevant regulations regarding the use of human study participants and was conducted in accordance with the criteria set by the Declaration of Helsinki. As we demonstrated previously [54], the human corneoscleral segments were divided into four equal quadrants and cultured separately in a 12-well plate using DMEM medium supplemented with 10% FBS, L-glutamine, and 1% Pen-strep. Each individual quadrant was treated with either ethanol (0.1%) or Dex (100 nM) or Dex (100 nM) plus PBA (5 mM) or Dex (100 nM) plus PBA (5 mM) plus MCHCl (200 µM) for 7 d. Following treatment, conditioned medium was collected for Western blot analysis and tissue quadrants were fixed in 4% paraformaldehyde and subjected to immunostaining analysis using appropriate antibodies.

### 4.9. Decellularization

As described previously [12], primary human TM cells were grown to 80% confluence on 4-well chamber slides and treated with either ethanol or Dex or Dex plus PBA or PBA or Dex plus PBA plus MCHCl for 7 d. Following the treatment, cells were detached by sequentially treating the wells with 0.2% Triton X-100 for 10 min at 37  °C and with 0.3% ammonium hydroxide solution (NH_4_OH) for 5 min at 37 °C. Following the complete decellularization process, untreated primary human TM cells were re-plated on the same 4-well chamber slides and treated with or without PBA (5 mM) and MCHCl (200 µM) for an additional 4–6 d.

### 4.10. Immunostaining

*Mouse and human tissue sections:* Enucleated mouse eyes and ex vivo cultured human corneoscleral segments were fixed in 4% paraformaldehyde for 3 h. The fixed eyes and tissue segments were processed and embedded in paraffin. Tissue sections with five-micron thickness were deparaffinized in xylene and rehydrated using gradient concentrations of ethanol (100–50%). Followed by antigen retrieval with citrate buffer (pH  6.0), tissue sections were treated with blocking buffer (PBS containing 10% goat serum and 0.2% Triton X-100) for 2  h at room temperature. Then, the tissue sections were incubated with specific primary antibodies at 4  °C for 16 h. Following 3 washes in PBS, sections were incubated with an appropriate Alexa fluor secondary antibodies for 2  h at room temperature. Later, tissue sections were washed thoroughly with PBS and mounted with mounting medium containing DAPI nuclear stain (Vector Labs, Inc., Burlingame, CA, USA).

#### Primary Human TM Cells

Primary human TM cells were fixed in 4% PFA for 15 min and washed thoroughly with PBS. Cells were incubated with blocking buffer (10% goat serum and 0.2% Triton-X-100 in PBS) for 2  h at room temperature, and then with appropriate primary antibodies for 16 h at 4 °C. Cells were washed with PBS, and then incubated with appropriate Alexa Fluor secondary antibodies (Life technologies, Grand Island, NY, USA). After three final washes in PBS, cells were mounted with DAPI-mounting solution.

Images were captured using either Leica confocal SP8 microscope (Buffalo Grove, IL, USA) or Keyence fluorescence microscope (Itasca, IL, USA) and the fluorescent intensities were analyzed using ImageJ software [55]. As described earlier [11], tissue sections or cells incubated without primary antibody served as a negative control and the images were captured by keeping constant laser exposure settings, adjusted to the negative control.

#### 4.11. Western Blot Analysis

Protein lysates were obtained by lysing the TM cells in radioimmunoprecipitation assay (RIPA) lysis buffer supplemented with complete protease and phosphatase inhibitors cocktail. For analyzing the secreted ECM proteins, conditioned media was concentrated and subjected to Western blot analysis. The total protein concentration in cellular lysates was estimated by Lowry method. Approximately 30 µg of protein lysates were subjected to polyacrylamide gel electrophoresis under denaturing conditions and then the resolved proteins were transferred to polyvinylidene difluoride (PVDF) membranes. Each blot was blocked with 10 ml of blocking buffer (10% non-fat dry milk) for 1 h at room temperature and then incubated with specific primary antibodies for 16 h at 4 °C. Blots were then washed with 1x PBST for 3 times and further incubated at room temperature for 2 h with corresponding secondary antibody, conjugated with horseradish peroxidase. Using chemiluminescence detection reagents, the blot was visualized and quantified by Odyssey Fc Imager [56]. Blots were probed with GAPDH as a loading control.

#### 4.12. Gelatin Zymography

Primary human TM cells or transformed GTM3 cells were treated with ethanol (0.1%) or Dex (100 nM) or Dex (100 nM) plus PBA (5 mM) for 3 to 4 d under serum free conditions. Followed by treatment, conditioned media was collected and concentrated using protein concentrators with 3 K MWCO. The concentrated conditioned media was subjected to gelatin zymography electrophoresis under native conditions. Following electrophoresis, gels were washed with washing buffer and incubated in zymogram renaturing buffer for 30 min at room temperature with gentle agitation. Gels were further incubated with zymogram developing buffer for 24 h at room temperature. Gels were stained for 1 h with staining solution at room temperature and later distained until the bands became visible.

#### 4.13. Quantitative Real Time Polymerase Chain Reaction (qPCR) Analysis

Transformed GTM-3 cells were treated with ethanol (0.1%) or Dex (100 nM) or Dex (100 nM) plus PBA (5 mM) or PBA (5 mM) for 24 h. Total RNA was isolated using RNeasy mini kit (Qiagen) as per the manufacturer’s instructions. Isolated RNA concentration and purity were measured by NanoDrop 2000 (Thermo Fisher Scientific). Later, cDNA was synthesized from the isolated RNA using SuperScript VILO cDNA synthesis kit (Thermo Fisher Scientific) and subjected to qPCR. We used 2× SsoAdvanced SYBR Green Supermix to perform qPCR reaction and BioRad CFX96 thermocycler. The PCR conditions included an initial 95 °C for 60 s incubation was followed by 30 cycles at 95 °C for 60 s, 60 °C for 45 s, and 72 °C for 45 s, and completed with a dissociation curve. The mRNA expression levels of MMP9 and TIMP1 genes were normalized to level of RPLP0. The primer sequences used in qPCR were as follows: human *MMP9* (forward 5′-CGAACTTTGACAGCGACAAG-3′ and reverse 5′-CACTGAGGAATGATCTAAGCCC), human *TIMP1* (forward 5′- TTCTGCAATTCCGACCTCG-3′ and reverse 5′-TCATAACGCTGGTATAAGGTGG) and *RPLPO* (forward 5′-TCGTCTTTAAACCCTGCGTG-3′ and reverse 5′-TGTCTGCTCCCACAATGAAAC).

#### 4.14. Statistical Analysis

Statistical analysis was performed by using GraphPad Prism software version 9.0 (GraphPad software, CA, USA) [57]. Data sets were analyzed using either one-way or two-way ANOVA with multiple comparisons and expressed as mean ± SD. A *p* value less than 0.05 was considered statistically significant.

## Data Availability

The data that support the findings of this study are available from the corresponding author upon reasonable request.
